# Comparison of anti-angiogenic properties of pristine carbon nanoparticles

**DOI:** 10.1186/1556-276X-8-195

**Published:** 2013-04-26

**Authors:** Mateusz Wierzbicki, Ewa Sawosz, Marta Grodzik, Marta Prasek, Slawomir Jaworski, André Chwalibog

**Affiliations:** 1 Division of Biotechnology and Biochemistry of Nutrition, Warsaw University of Life Sciences, Warsaw, Poland; 2 Department of Veterinary Clinical and Animal Sciences, University of Copenhagen, Groennegaardsvej 3, Frederiksberg, Copenhagen, 1870, Denmark

**Keywords:** Carbon nanoparticles, Angiogenesis, Graphene, Graphite, Fullerene, Diamond, Nanotubes

## Abstract

Angiogenesis is vital for tumour formation, development and metastasis. Recent reports show that carbon nanomaterials inhibit various angiogenic signalling pathways and, therefore, can be potentially used in anti-angiogenic therapy. In the present study, we compared the effect of different carbon nanomaterials on blood vessel development. Diamond nanoparticles, graphite nanoparticles, graphene nanosheets, multi-wall nanotubes and C60 fullerenes were evaluated for their angiogenic activities using the *in ovo* chick embryo chorioallantoic membrane model. Diamond nanoparticles and multi-wall nanotubes showed the greatest anti-angiogenic properties. Interestingly, fullerene exhibited the opposite effect, increasing blood vessel development, while graphite nanoparticles and graphene had no effect. Subsequently, protein levels of pro-angiogenic growth factor receptors were analysed, showing that diamond nanoparticles decreased the expression of vascular endothelial growth factor receptor. These results provide new insights into the biological activity of carbon nanomaterials and emphasise the potential use of multi-wall nanotubes and diamond nanoparticles in anti-angiogenic tumour therapy.

## Background

Angiogenesis is the most common process of new blood vessel development. Growth of new vessels starts from pre-existing ones and consists of two main processes: sprouting (endothelial cell migration) and intussusception (splitting of vessels) [1,2]. The growth of blood vessels depends on a balance between angiogenesis-promoting and angiogenesis-inhibiting signalling molecules. Vascular network growth is an essential process, especially during embryonic development, tissue remodelling and regeneration. However, disorders in blood vessel development may foster diseases like chronic inflammatory disorders. Development of new vessels is also essential for the growth and metastasis of tumours, in which pro-angiogenic molecules like vascular endothelial growth factor (VEGF) and fibroblast growth factor (FGF) play critical roles. Binding of FGF and especially VEGF, which is considered a major molecule controlling blood vessel morphogenesis, to their tyrosine kinase receptors activates multiple downstream molecules involved in different signalling pathways that lead to increased vascular permeability, cell migration and proliferation [3]. The VEGF receptor KDR (also called Flk-1) and the FGF receptor FGFR are responsible for regulating angiogenesis (for reviews, see [4,5]).

Therapeutic anti-angiogenic compounds have been extensively studied for anti-tumour therapy. VEGF inhibitors have been approved for clinical use in cancer diseases. However, anti-VEGF therapy is effective only in particular cases and can lead to serious toxicity [6,7]. Angiogenesis is a complex process regulated by several regulators. Inhibiting only the VEGF signalling pathway seems to be insufficient. Hence, therapeutic agents affecting tumour cells without harming healthy cells are necessary to optimise cancer treatments. Carbon nanomaterials can be used as low-toxicity inhibitors of tumour angiogenesis. It has been demonstrated that nanoparticles of diamond, graphite, graphene, nanotubes and fullerenes display low toxicity [8-11]. Recently, we showed that diamond nanoparticles and microwave-radiofrequency carbon decreased the vascular network in glioblastoma tumours and mRNA levels of VEGFA and bFGF [12]. Furthermore, because of their high surface-to-volume ratio, carbon nanomaterials cause high biological activity and enable easy surface modification [13,14]. We hypothesised that pristine carbon nanoparticles can affect VEGF and bFGF receptors and inhibit tumour angiogenesis, but the effectiveness of anti-angiogenic activity can vary between different carbon nanostructures. Consequently, the objective of this study was to explore the anti-angiogenic properties of different carbon nanomaterials to find the most efficient for anti-angiogenic tumour therapy.

## Methods

### Nanomaterials

**Figure 1:**
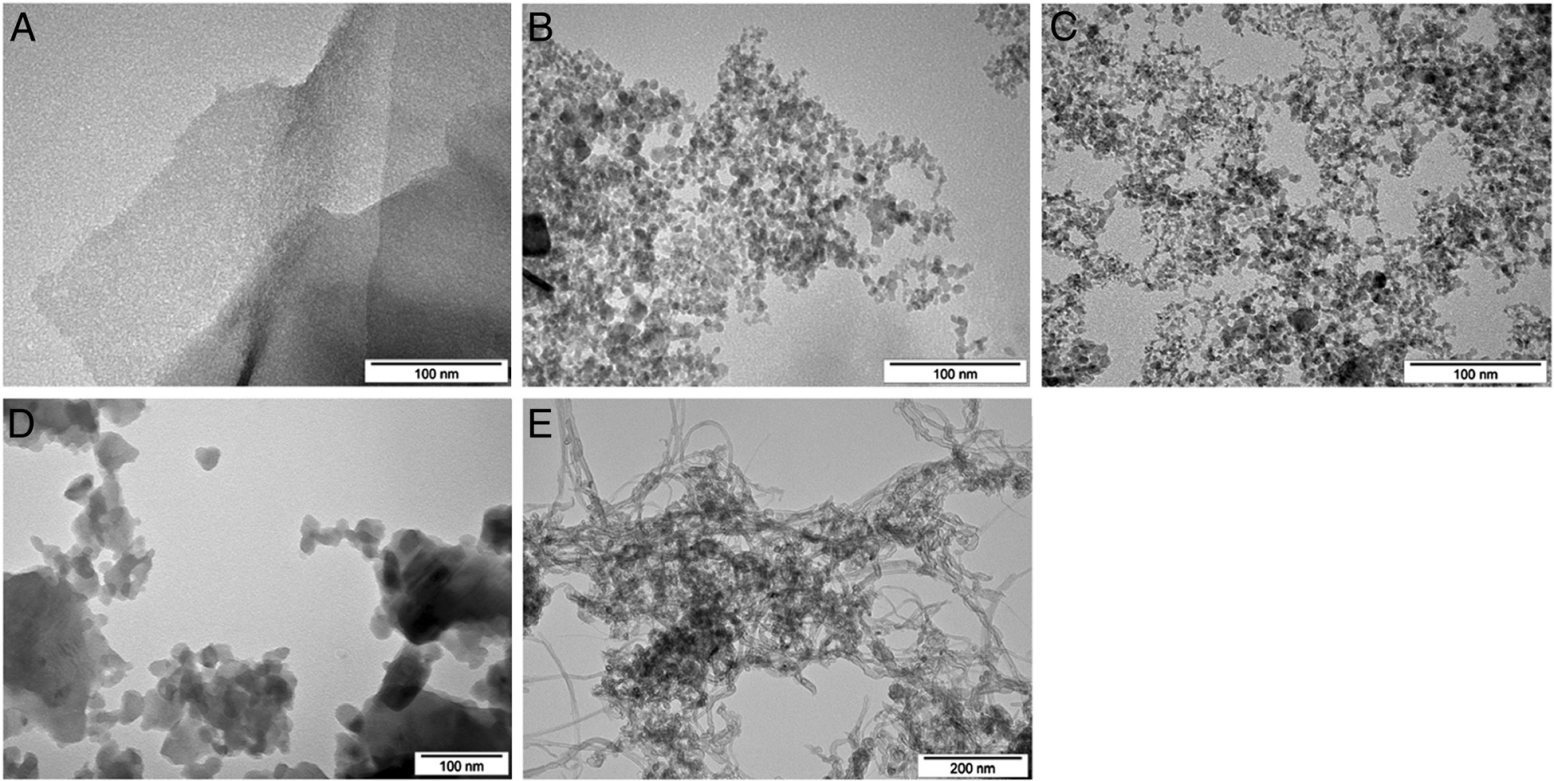
**Transmission electron microscopic images of carbon nanomaterials.** (**A**) GNS, (**B**) NG, (**C**) ND, (**D**) C60 and (**E**) MWNT.

In the present study, we used *in ovo* chicken embryo chorioallantoic membranes (CAM) to compare the anti-angiogenic properties of pristine carbon nanomaterials: diamond nanoparticles (ND), graphite nanoparticles (NG), graphene nanosheets (GNS), multi-wall nanotubes (MWNT) and C60 fullerenes (C60). The physical characteristics of the nanoparticles are given in Table 1. ND and NG are spherical nanoparticles, produced by the detonation method with size ranging from 3 to 4 nm. C60 is a spherical nanoparticle that in water solvent aggregates into particles with a mean size of approximately 50 nm. GNS and MWNT are nanomaterials having diameters of 6 to 8 nm and 8 nm, and length of approximately 15 μm and 5 to 20 μm, respectively. Purity and specific surface area (except C60) were provided by the manufacturers. C60 was obtained from SES Research (Houston, TX, USA), and all other materials were from Skyspring Nanomaterials (Houston, TX, USA). The nanomaterials were dispersed in demineralised water using sonication. New solutions were made a day before each repetition. The shape and size of the nanomaterials were visualised using a JEM-2000EX transmission electron microscope (JEOL Ltd., Tokyo, Japan) at 200 kV (Figure 1). Zeta potential measurements were carried out on a Zetasizer Nano-ZS90 (Malvern, Worcestershire, UK) at 25°C. Measurements were conducted using Smoluchowski approximation, which is rigorously valid only for spherical particles. Therefore, the zeta potential of non-spherical nanomaterials may be overestimated by up to 20% [15].

**Table 1:** Comparison of the physical characteristics of the carbon nanoparticles

	**GNS**	**NG**	**ND**	**C60**	**MWNT**
Shape	Nanosheet	Spherical nanoparticle	Spherical nanoparticle	Spherical nanoparticle	Multi-wall tube
Size	6 to 8 nm/15 μm	3 to 4 nm	3 to 4 nm	Approximately 50 nm (aggregates)	8 nm/5 to 20 μm
Atom configuration	*sp*^2^	*sp*^2^	*sp*^3^	*sp*^2^	*sp*^2^
Purity	>99.5%	>93%	>95%	>99.5%	>95%
Zeta potential (mV)	−3.83	28.7	−39.3	−38.0	−14.8
Specific surface area	120 to 150 m^2^/g	540 to 650 m^2^/g	Approximately 282 m^2^/g	0.07 to 0.17 m^2^/g^a^	>500 m^2^/g
Production	Exfoliated	Explosion	Explosion	Arc discharge	Catalytic CVD

Purity and specific surface area (except C60) were provided by the manufacturer. The size was provided by the manufacturer and examined with a transmission electron microscope. Zeta potential was examined with Zetasizer Nano-ZS90. GNS, graphene nanosheet; NG, graphite nanoparticle; ND, diamond nanoparticle; C60, fullerene C60; MWNT, multi-wall nanotube; CVD, chemical vapour deposition. ^a^Taken from Cheng et al. [16].

### CAM assay

CAM implants were made from sterile Waterman filter paper with a diameter of 10 mm. Water (control) or hydrocolloids of nanoparticles of a concentration of 500 mg/L were added to the implants (final amount of nanoparticles on the implant was 0.01 mg). The implants were pre-treated with 3 mg/mL of hydrocortisone sodium succinate (Sigma, St. Louis, MO, USA) and air dried under sterile conditions. Fertilised eggs from Ross line 308 hens were obtained from a certified hatchery and kept for 4 days at 12°C. The eggs were cleaned, sterilised with UVC light and divided into six groups (6 × 20 eggs). Embryos were incubated at standard conditions (temperature 37°C, humidity 60% and turned once per hour). Embryonic day 0 (E0) started when the eggs were placed into the incubator. At day E6, small holes (1 cm^2^) were made on the shell above air space, the inner membrane was gently stripped off, and the implants were placed on CAM. The chicken embryos were incubated until day 7 of embryonic development, when implants with CAM were prefixed with 1.5 mL of 4% paraformaldehyde. After 30 min of incubation at 4°C, CAM with implants were cut out and fixed at 4°C in 4% paraformaldehyde for 60 min (total fixation time, 90 min). After fixation, the implants were gently stripped off. All measurements were repeated three times minimum.

### CAM tissue angiogenesis analysis

**Figure 2:**
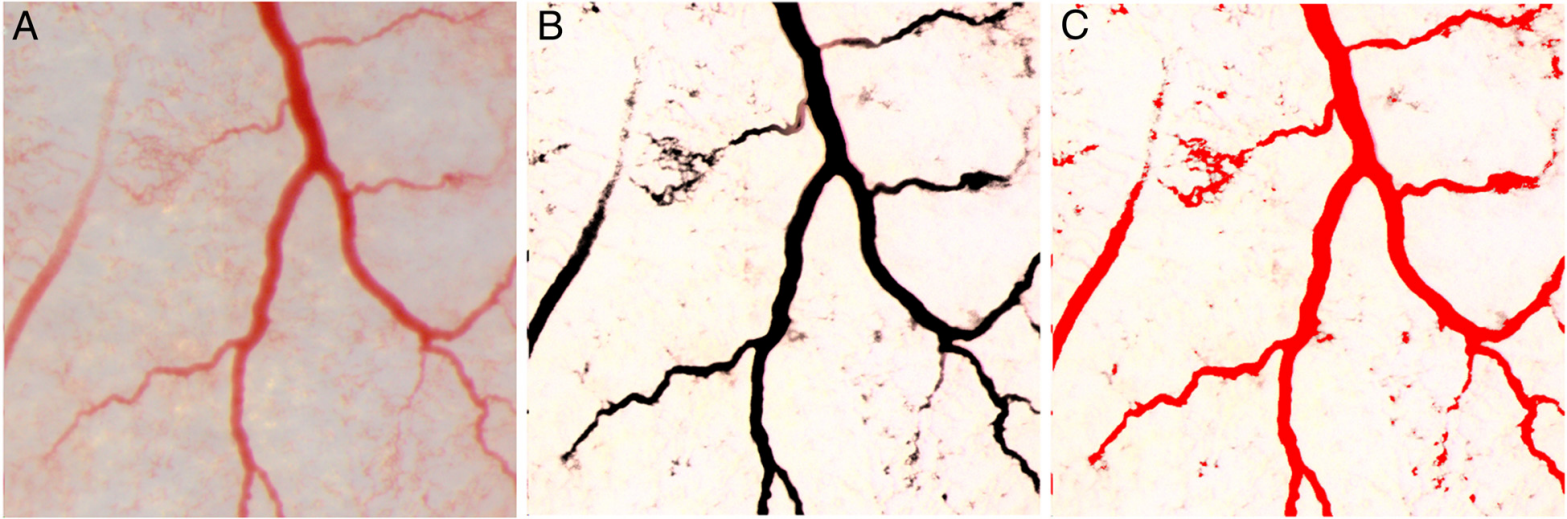
**CAM assay for determining total area of vessels with CellSens Dimension Desktop version 1.3.** (**A**) CAM square area of dimensions 2.5 × 2.5 mm and (**B**) image with a strengthened local contrast of images by increasing intensity and brightness. (**C**) For total area calculation, the threshold was set at intensity volumes between 0 and 256 for the shades of red, 0 and 256 for green, 0 and 145 and for blue.

The methodology of quantifying blood vessel development was based on [17] and [18], validated and used for this investigation. CAM tissues from implants were investigated with a stereomicroscope under a 12.5-fold magnification (SZX10, CellD software version 3.1, Olympus Corporation, Tokyo, Japan). Photos were analysed with CellSens Dimension Desktop version 1.3 (Olympus Corporation). The level of angiogenesis in eight CAM tissues from each group was determined by calculating the vessel area, length and number of branch points on three square areas of dimensions 2.5 × 2.5 mm (total area, 18.75 mm^2^ out of 78.5 mm^2^). CAM tissue areas were selected semi-randomly so that the vessels with a diameter greater than 200 μm were not assessed. Vessel area, length and number of branch points were calculated separately for vessels with a diameter smaller than 100 μm and those between 100 and 200 μm. To calculate the vessel area, the intensity differences between vessels and background were increased. Local contrast of images was strengthened by increasing the intensity by 20 and brightness by 300 (kernel radius, 128). The threshold was set at intensity volumes between 0 and 256 for shades of red, 0 and 256 for green, and 0 and 145 for blue (Figure 2).

### CAM tissue morphological analysis

CAM implant morphology and development of capillary vessels were determined with the stereomicroscope described above. CAM cross sections were made with a cryostat (CM 1900, Leica, Wetzlar, Germany). Blocks were cut into 5-μm-thick sections and observed under a light microscope (DM 750, Leica).

### Immunoblotting

Protein levels of CAM KDR and FGFR were examined by Western blot analysis. Protein extracts were prepared with TissueLyser LT (Qiagen, Hilden, Germany) using ice-cold RIPA buffer (150 mM NaCl, 0.5% sodium deoxycholate, 1% NP-40, 0.1% SDS, 50 mM Tris, pH 7.4) with protease and phosphatase inhibitors (Sigma). The protein concentration was determined by the Total Protein Kit, Micro Lowry, Peterson's Modification (Sigma). An equal volume (50 mg) of samples was denatured by the addition of sample buffer (Bio-Rad Laboratories, Munich, Germany) and boiled for 4 min. Proteins were resolved under reductive conditions with SDS-PAGE and transferred onto PVDF membrane (Life Technologies, Gaithersburg, MD, USA). Protein bands were visualised with the GelDoc scanner (Bio-Rad Laboratories), using the fluorescent method of the WesternDot Kit (Life Technologies) and the primary antibodies bGFR (cat. no. F4305-08, USBiological, Swampscott, MA, USA), KDR (cat. no. SAB4300356, Sigma) and GAPDH (cat. no. NB300-327, Novus Biologicals, Cambridge, UK) as loading control (dilutions recommended by the producers). Protein bands were characterised using the Quantity One 1-D analysis software (Bio-Rad Laboratories).

### Statistical analysis

A one-way analysis of variance with the Bonferroni *post hoc* test was used for multiple comparisons. Differences at *P* < 0.05 were considered significant. Results are shown as means and standard errors.

## Results

**Figure 3:**
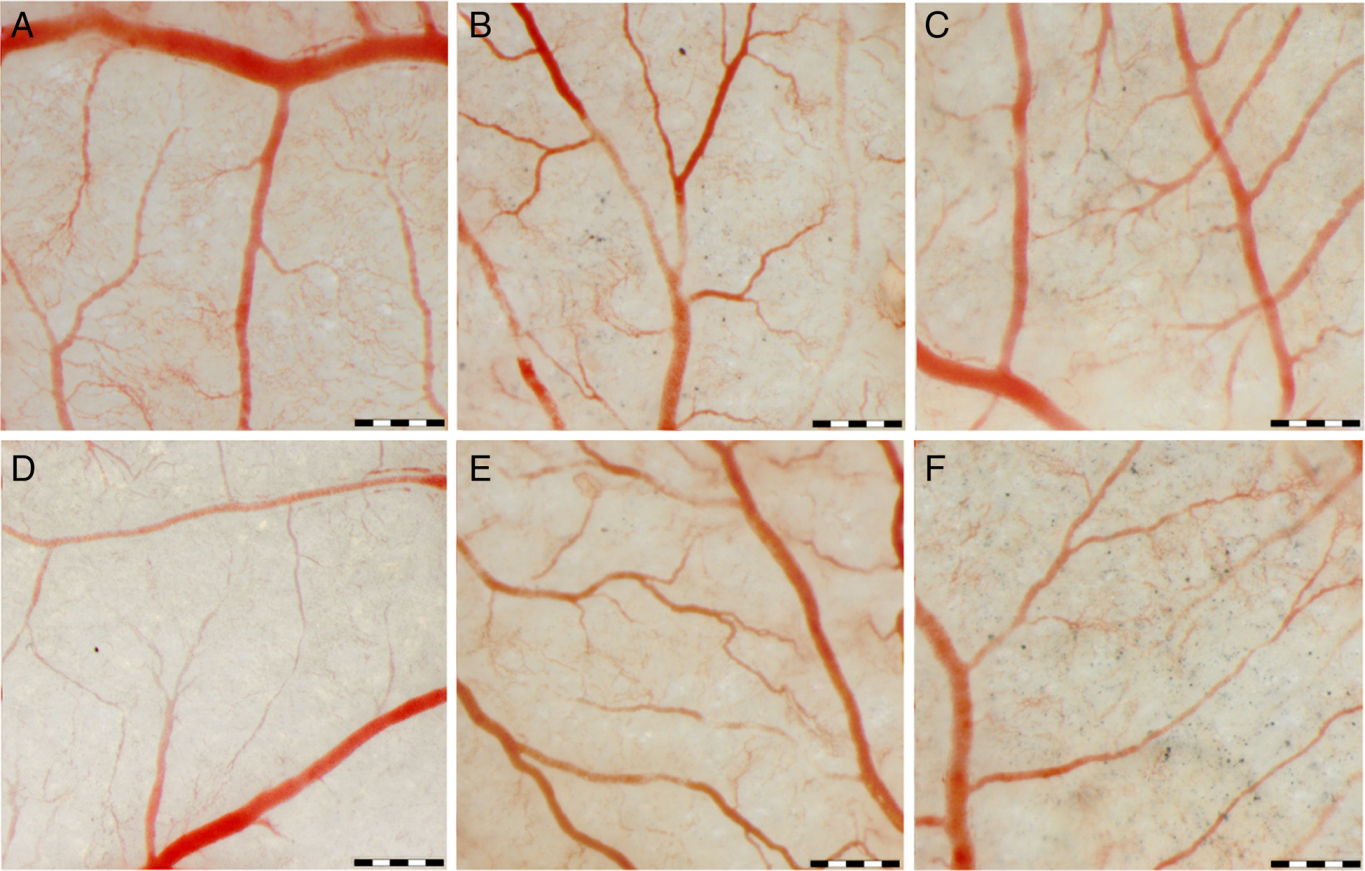
**CAM vessel morphology in response to treatment with carbon nanoparticles.** (**A**) Control, (**B**) GNS, (**C**) NG, (**D**) ND, (**E**) C60 and (**F**) MWNT. Scale bar, 500 μm.

We compared the influence of different carbon nanoparticles on the development of blood vessels, using the chicken embryo CAM implantation method as a model for angiogenesis [19]. The experiments were repeated three times minimum, and all repetitions gave equivalent results. Changes in the development of blood vessels after nanoparticle treatments were observed by measuring changes in the mean vessel area, vessel length and the number of branch points. These parameters were investigated in vessels at two development states: older with a diameter between 100 and 200 μm and newly developed with a diameter smaller than 100 μm. The area of blood vessels with a diameter between 100 and 200 μm was the largest in the C60-treated group. However, these changes were not statistically significant (Table 2). Vessel length decreased after MWNT and ND treatment. Both nanoparticles caused a comparable decrease in blood vessel length. Of all the investigated nanoparticles, only ND significantly decreased the number of branch points. Assessment of the development of vessels with a diameter smaller than 100 μm showed different results. The area of newly developed vessels treated with ND was significantly smaller, compared to the other groups (Table 3). Both ND and MWNT decreased vessel length and the number of branch points, but ND had a significantly stronger effect. Furthermore, capillary vessels of MWNT- and especially ND-treated implants were poorly developed (Figure 3). Vessel branching was also affected by C60, resulting in an increased number of vessel branch points. NG and GNS showed no effect on the examined parameters in both older and newly formed vessels.

**Table 2:** Comparison of angiogenesis parameters of vessels with a diameter between 100 and 200 μm

	**Mean vessel area (mm**^**2**^**)**	**Mean vessel length (mm)**	**Number of branch points**	**Angiogenic activity**
Group				
Control	30.8	2.8 a	4.3 a	
GNS	26.9	2.3 ab	4.2 ab	0
NG	24.9	2.0 ab	2.6 ab	0
ND	25.9	1.9 b	1.8 b	- -
C60	39.4	2.7 a	3.8 ab	0
MWNT	25.4	1.7 b	3.4 ab	- -
*P* value	0.038	0.006	0.014	
Pooled SE	3.5	0.2	0.5	

A 0 means no activity, a hyphen indicates low anti-angiogenic activity, and two hyphens indicate medium anti-angiogenic activity. Values with different letters are significantly different, *P* < 0.05. SE, standard error; GNS, graphene nanosheets; NG, graphite nanoparticle; ND, diamond nanoparticle; C60, fullerene C60; MWNT, multi-wall nanotube.

**Table 3:** Comparison of angiogenesis parameters of vessels with a diameter less than 100 μm

	**Mean vessel area (mm^2^)**	**Mean vessel length (mm)**	**Number of branch points**	**Angiogenic activity**
Group				
Control	44.9 a	9.9 a	11.4 a	
GNS	50.0 a	9.9 a	13.4 ab	+ (tendency)
NG	47.5 a	9.1 ab	10.1 a	0
ND	33.1 b	7.7 b	5.5 c	- -
C60	52.1 a	11.9 c	14.7 b	+++
MWNT	46.2 a	8.7 ab	9.1 d	- -
*P* value	0.004	0.000	0.000	
Pooled SE	3.1	0.4	0.7	

A 0 means no activity; a plus sign indicates low pro-angiogenic activity; three plus signs indicate high pro-angiogenic activity; two hyphens indicate medium anti-angiogenic activity; and three hyphens indicate high anti-angiogenic activity. Values with different letters are significantly different, *P* < 0.05. SE, standard error; GNS, graphene nanosheet; NG, graphite nanoparticle; ND, diamond nanoparticle; C60, fullerene C60; MWNT, multi-wall nanotube.

**Figure 4:**
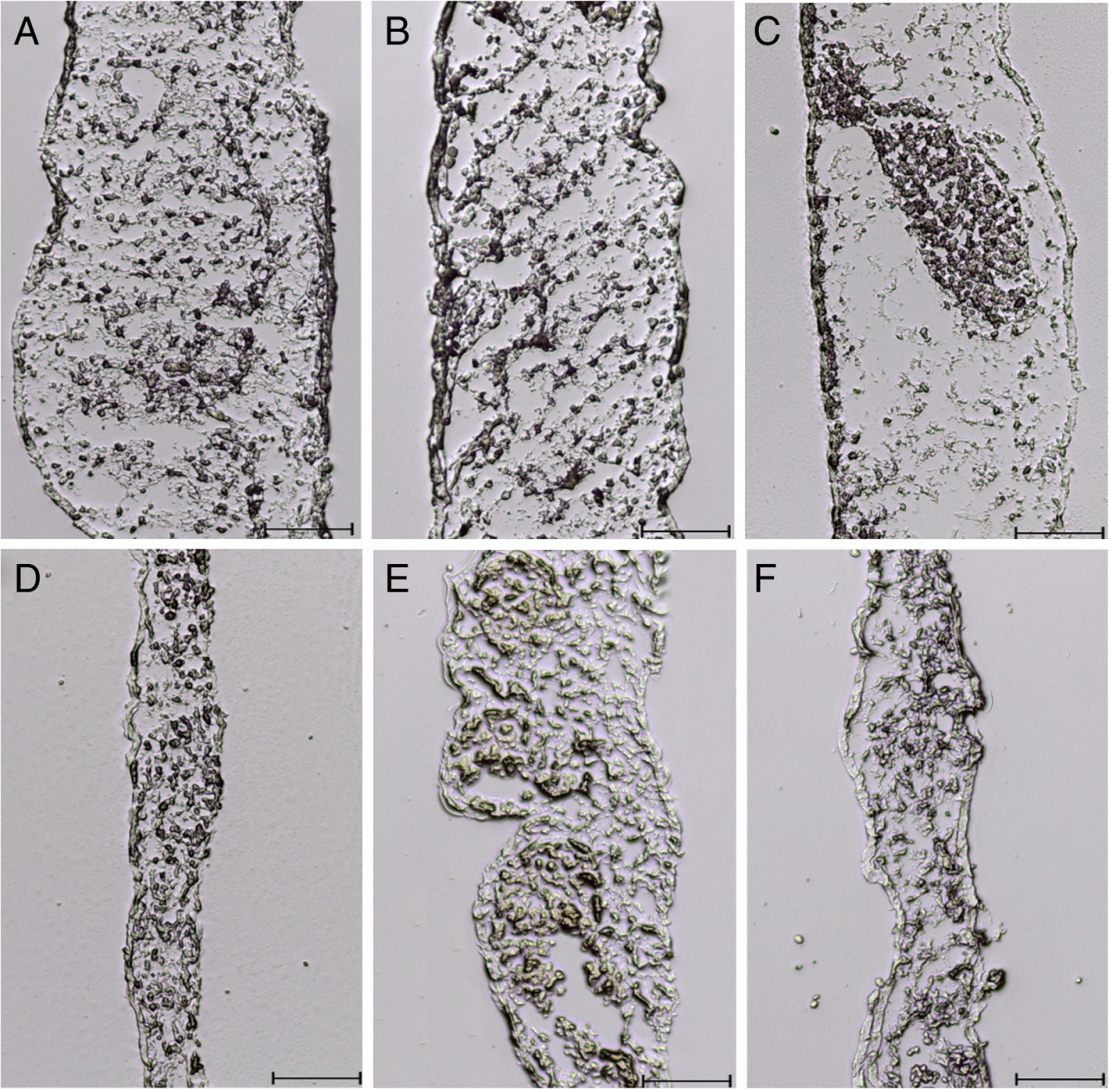
**Cross sections of CAM tissue treated with carbon nanoparticles.** (**A**) Control, (**B**) GNS, (**C**) NG, (**D**) ND, (**E**) C60 and (**F**) MWNT. Scale bar, 100 μm.

To confirm whether nanoparticles affected CAM morphology, we investigated CAM cross sections (Figure 4). In the control group, the mean CAM thickness varied between 250 and 380 μm. In the ND- and MWNT-treated groups, the mean thickness varied between 80 and 200 μm and 90 and 260 μm, respectively. The other tested nanoparticles did not affect CAM morphology.

**Figure 5:**
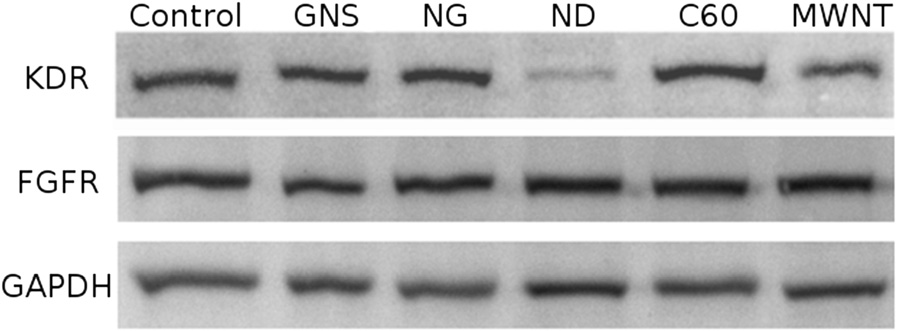
**Representative immunoblot of KDR and FGFR CAM protein expression levels examined by Western blotting.** GNS, graphene nanosheet; NG, graphite nanoparticle; ND, diamond nanoparticle; C60, fullerene C60; MWNT, multi-wall nanotube; KDR, vascular endothelial growth factor receptor; FGFR, fibroblast growth factor receptor; GAPDH, glyceraldehyde-3-phosphate dehydrogenase.

Expression of KDR correlated with the pro- and anti-angiogenic properties of C60 and ND, but not MWNT (Table 4, Figure 5). Compared to the control group, ND reduced the expression of KDR by 38%. Fullerenes increased the KDR protein level by 30%. The other tested nanoparticles did not significantly alter the protein levels of KDR. FGFR protein amounts were not affected by all the tested carbon nanoparticles.

**Table 4:** Relative percentage of KDR and FGFR protein levels calculated with GAPDH as the loading control

**Protein**	**Groups**						**ANOVA**	
	**Control (%)**	**GNS (%)**	**NG (%)**	**ND (%)**	**C60 (%)**	**MWNT (%)**	***P*value**	**Pooled SE**
KDR	100.0 a	102.1 a	103.3 a	62.0 b	129.6 c	102.7 a	0.000	2.4
FGFR	100.0 ab	96.0 a	103.6 ab	95.3 a	108.3 b	104.2 ab	0.000	2.0

Values with different letters are significantly different, *P* < 0.05. ANOVA, analysis of variance; SE, standard error; GNS, graphene nanosheet; NG, graphite nanoparticle; ND, diamond nanoparticle; C60, fullerene C60; MWNT, multi-wall nanotube; KDR, vascular endothelial growth factor receptor; FGFR, fibroblast growth factor receptor; GAPDH, glyceraldehyde-3-phosphate dehydrogenase.

## Discussion

In this work, we compared the anti-angiogenic properties of carbon-based nanomaterials. The measurements were performed using the well-established chicken embryo CAM model [17,19]. CAM growth is essential for embryo development and is almost complete by 1 to 14 days of embryogenesis [20]. Blood vessel development was examined in the phase of the most intensive vessel growth, which was on day 7 of chicken embryo development [21].

The present results are consistent with our previous research, demonstrating that ND and microwave-radiofrequency carbon allotrope decreased the vascular network in glioblastoma tumour and, consequently, their volume and weight. Moreover, diamond nanoparticles decreased the mRNA level of the main pro-angiogenic factors VEGFA and bFGF [12]. ND also affected the transcription level of the human stress-responsive genes of cells exposed to stress (heat shock, cytotoxic and oxidative stress). It has been demonstrated that although ND did not show toxic effects on leukaemia cell line HL-60, it up-regulates the expression of the gene SOD1, responsible for the defence mechanism against reactive oxygen species, and down-regulates the genes JUN, GADD45A and FRAP1, responsible for protection against genotoxic and cellular stress [22]. Moreover, the anti-angiogenic activity of nanoparticles has been related to their inhibitory effects on pro-angiogenic factors. Gold nanoparticles specifically bind to VEGFA and bFGF and inhibit their interaction with cell membrane receptors [23,24].

Among all the tested nanoparticles, only MWNT and more significantly ND showed anti-angiogenic activity. Nanomaterials with graphite structure (NG and GNS) did not alter blood vessel development. There are only a few studies on the biological activity of GNS. Wang et al. [25] showed that GNS oxide exhibited low toxicity in mice and human fibroblast cells. Furthermore, GNS displayed low cytotoxicity in erythrocytes and fibroblasts [26], which together with our results suggests that GNS is highly biocompatible with the vascular system. Similarly, NG had no effect on CAM angiogenesis, although they have the same shape and similar size and are produced in the same way (but under different conditions) as ND [27], which had the strongest anti-angiogenic activity (Table 1). The strongest inhibition of vessel growth by ND may be linked to the inhibition of VEGF receptor (KDR) expression. VEGF is a major pro-angiogenic factor essential for the development of the blood vessel network. It is controlled by the release of growth factors dependent on the oxygen level, with HIF-1 being one of the most important [3]. Hypoxia leads to the up-regulation of VEGF and, thus, the formation of new blood vessels, which consequently normalises the oxygen status. In tumours, high activity and fast divisions of tumour cells lead to oxygen deficiency that enhances vessel growth. KDR is also regulated by various signalling molecules in response to changes in oxygen concentration [28,29]. Hypoxia leads to KDR up-regulation and activation of the angiogenic signalling cascade [30,31]. Down-regulation of KDR by ND may decrease hypoxia-mediated angiogenesis and exert efficient and long-lasting anti-angiogenic effects. Moreover, chronic hypoxia can lead to further down-regulation of KDR [32]. MWNT showed anti-angiogenic activity, inhibiting the branching of vessels with a diameter smaller than 100 μm. This indicates that MWNT inhibits the development of smaller/younger vessels only. Our report is consistent with the results of another study showing that pristine MWNT displayed an anti-angiogenic effect on an *in vivo* VEGFA/bFGF-induced model [33] and in *in vitro* HUVEC tubule formation assays [34]. However, doxorubicin conjugated with single-wall nanotubes had the opposite effects [35].

As expected, nanoparticles had less impact on the development of older vessels. Only ND, which exerted the strongest anti-angiogenic properties, induced a significant decrease in vessel length and the number of branch points. However, ND did not change the area of older vessels (100 to 200 μm). Reduced length and branching without significant changes in vessel area suggest that ND can inhibit the development of vessels with dimensions that slightly exceed 100 μm and smaller. The present results give new insights into the bioactive properties of ND and clearly show that this carbon nanoparticle can be considered for use in low-toxicity anti-angiogenic therapy.

Interestingly, our results demonstrated pro-angiogenic activity of pristine C60, which increased the number of branch points and vessel length. Fullerene C60 has been used to inhibit cancer growth [36] and is used as photosensitisers in photodynamic therapy [37]. However, Zogovic et al. [38] studied the effect of nanocrystaline fullerene on melanoma tumour and showed that fullerene, probably by immunosuppression, had tumour-promoting activity and increased the production of nitric oxide (NO), which can promote angiogenesis [39]. Furthermore, other reactive oxygen species can also induce angiogenesis [40]. The ability of C60 to generate reactive oxygen species has been previously demonstrated [41,42]. NO promotes angiogenesis by up-regulating the expression of the VEGFA receptor [43], which is consistent with our report. This appears to be the most probable mechanism underlying fullerene pro-angiogenic effects and may only be specific for pristine nanoparticles. Hydroxylated C60 has been shown to protect cells *in vitro* form oxidative stress, while pristine nanoparticles show pro-oxidant capacity [44,45]. Moreover, C60 modified with multihydroxylated metal can simultaneously down-regulate more than ten angiogenic factors and significantly decrease the capillary vessels of tumours (average size 1.2 cm in diameter) [46]. Murugesan et al. [33] demonstrated that pristine MWNT and C60 inhibited the angiogenesis induced by exogenous VEGFA or bFGF. Our results indicated that C60 had the opposite effect on vessels not stimulated by exogenous pro-angiogenic factors. This suggests that C60 can have both anti- and pro-angiogenic activity depending on the physiological state of blood vessels.

## Conclusions

We compared the anti-angiogenic properties of pristine carbon nanomaterials. According to our results, the carbon nanomaterials showing the most anti-angiogenic to pro-angiogenic properties were as follows: diamond nanoparticles (anti-angiogenic) - multi-wall nanotubes - graphite nanoparticles (no activity) - graphene nanosheets - fullerene C60 (pro-angiogenic). Only diamond nanoparticles, multi-wall nanotubes and fullerenes showed statistically significant results. Nanoparticles showing anti-angiogenic effects also changed the morphology of CAM by decreasing its thickness. Diamond nanoparticles and fullerene changed the expression level of KDR, but not FGFR, thereby affecting the angiogenic potential of CAM. Multi-wall nanotubes and especially diamond nanoparticle can be considered potential inhibitors of blood vessel growth in anti-angiogenic tumour therapy.

## Competing interests

The authors declare that they have no competing interests.

## Authors' contributions

MW prepared the angiogenesis assay, carried out the experimental analysis and drafted the manuscript. MW and MG performed the *in ovo* experiments. SJ made the TEM observations. MP carried out the immunobloting experiments. AC and ES supervised the work and finalized the manuscript. All authors read and approved the final manuscript.
